# Evaluating the Causal Effects of Serum and Cerebrospinal Fluid Metabolites on Cerebral Palsy: A Whole‐Metabolome Mendelian Randomization Study

**DOI:** 10.1002/brb3.70864

**Published:** 2025-09-21

**Authors:** Yonggang Dai, Wei Wang, Hongya Wang, Xuewei Zhuang

**Affiliations:** ^1^ Department of Clinical Laboratory Shandong Provincial Third Hospital Shandong University Jinan Shandong P. R. China; ^2^ Department of Clinical Microbiology Laboratory Shandong Second Provincial General Hospital Jinan Shandong P. R. China

**Keywords:** blood metabolites | causal effect | cerebral palsy | cerebrospinal fluid | Mendelian randomization

## Abstract

**Purpose:**

This study aimed to investigate the causal relationships between serum and cerebrospinal fluid (CSF) metabolites and cerebral palsy (CP) risk, leveraging genetic insights to identify potential biomarkers and metabolic pathways implicated in CP pathogenesis.

**Method:**

A two‐sample Mendelian randomization (MR) approach was employed to analyze 1400 serum metabolites and 338 CSF metabolites. Genetic variants associated with metabolite levels were used as instrumental variables (IVs) to infer causal effects on CP risk.

**Findings Serum Metabolites:**

Sixty‐nine metabolites showed significant associations with CP risk, including 1‐(1‐enyl‐stearoyl)‐2‐linoleoyl‐GPE (protective effect: odd ratio [OR] = 0.84, *p* = 0.001) and 1,2‐dipalmitoyl‐GPC (risk effect: OR = 1.12, *p* = 0.003). *CSF metabolites*: 13 metabolites were significantly linked to CP, most notably 1‐palmitoyl‐2‐palmitoleoyl‐GPC (OR = 0.57, *p* = 0.001). *Shared biomarker*: Methionine sulfone exhibited protective effects in both serum and CSF. *Pathway analysis*: Glyoxylate/dicarboxylate metabolism and butyrate metabolism emerged as key pathways potentially influencing CP pathogenesis.

**Conclusion:**

This MR study provides novel evidence supporting the causal role of serum and CSF metabolites in CP, highlighting methionine sulfone and specific metabolic pathways as biologically significant factors. Although limitations such as sample size constraints and lack of experimental validation warrant caution, these findings underscore the therapeutic potential of targeting metabolic pathways in CP. Future research should prioritize mechanistic studies and translational exploration of identified metabolites.

## Graphical abstract

A Mendelian randomization study identified 69 serum and 13 CSF metabolites associated with cerebral palsy risk, highlighting methionine sulfone as a shared protective biomarker and implicating glyoxylate, dicarboxylate, and butyrate metabolism in disease pathogenesis.

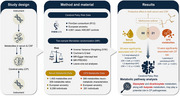

**Abbreviations**
CI: confidence interval
CP: cerebral palsy
CSF: cerebrospinal fluid
GWAS: genome‐wide association study
IVs: instrumental variables
IVW: inverse variance weighted
KEGG: Kyoto Encyclopedia of Genes and Genomes
MR: Mendelian randomization
ORs: odd ratios
SMPDB: Small Molecule Pathway Database
SNP: single nucleotide polymorphism

## 1 Introduction

Cerebral palsy (CP) is the most prevalent motor disability in children, affecting approximately one in 345 children globally (Zaman et al. 2025). It results from nonprogressive brain injury during development, leading to persistent disorders of movement and posture (Palanisamy et al. 2024). In accordance with the definition proposed by Rosenbaum et al. (2007), CP is also characterized by limitations in activity—defined as the execution of tasks or actions—and restrictions in participation—defined as involvement in life situations—which together reflect the broader impact of the condition on an individual's daily functioning and social integration (Palisano et al. 2008; Eliasson et al. 2006; Hidecker et al. 2011; Rosenbaum and Gorter 2012). Beyond its significant psychological and economic burdens, CP severely impacts children's quality of life and social adaptation (Trabacca et al. 2016). Despite advances in clinical characterization and neuroanatomical studies, the molecular mechanisms underlying CP remain poorly understood, creating a substantial gap in early diagnosis and effective therapeutic target identification (Reid et al. 2012).

Recent advances in metabolomics, a comprehensive approach to studying metabolites in biological samples, have provided insights into various neurological disorders, including stroke, Alzheimer's disease, and Parkinson's disease (Cryan et al. 2020). Emerging evidence suggests that metabolic dysregulation in CP may arise from aberrant DNA hypermethylation of promoter regions in genes governing energy metabolism, neuronal development, and membrane transport (Yuan 2017). Additionally, transcriptomic analyses have identified altered expression of energy metabolism‐related genes (e.g., GOT1, LPL) in CP muscle tissue, potentially contributing to impaired mitochondrial function and neuromuscular pathophysiology (Zheng et al. 2024). However, the causal relationship between serum and cerebrospinal fluid (CSF) metabolites and CP remains unexplored. Metabolic alterations may reflect disease‐specific biological processes, offering new avenues for early diagnosis and therapeutic intervention (Zahoor et al. 2021).

This study employed a two‐sample Mendelian randomization (MR) design using genetic variants as instrumental variables (IVs) to evaluate the causal effects of serum and CSF metabolites on CP risk. The MR approach minimizes confounding and reverse causality biases inherent in observational studies, providing robust causal inferences (Grover et al. 2017; Haycock et al. 2016; Davey Smith and Hemani 2014). By systematically analyzing serum and CSF metabolites, this study aimed to identify potential CP biomarkers and explore their roles in CP pathogenesis. Understanding these metabolic changes may not only elucidate disease mechanisms but also facilitate the development of novel diagnostic and therapeutic strategies (Huynh et al. 2022; Mu et al. 2024; Lee and Kim 2017).

## 2 Materials and Methods

### 2.1 Study Design

A two‐sample MR design was employed to assess the causal effects of metabolite‐associated genetic variants on CP susceptibility. Genetic variants associated with metabolites were used as IVs to infer causality. This approach allowed the use of genome‐wide association study (GWAS) summary statistics for metabolites and CP separately, under the assumption that these genetic variants were associated with both the exposure (metabolites) and the outcome (CP) (Hait and Stoffels 2021). The key assumptions of MR included

1. The genetic instruments were significantly associated with the metabolites (Panyard et al. 2021).
2. The IVs were not associated with any confounding factors (Melo et al. 2023).
3. The genetic variants influenced CP risk solely through their effects on metabolites, rather than via alternative pathways (Melo et al. 2023; Yang et al. 2020).

### 2.2 Data Sources

#### 2.2.1 Serum Metabolites

GWAS data for plasma metabolites were obtained from a study conducted by Chen et al., which included 8299 individuals of European ancestry (identifiers: GCST90199621 to GCST90201020) (Chen et al. 2023). This study analyzed 1091 metabolites and 309 metabolite ratios (Table S1). The metabolites were categorized into eight groups: lipids (395), amino acids (210), xenobiotics (130), nucleotides (33), cofactors and vitamins (31), carbohydrates (22), peptides (21), and energy metabolism‐related metabolites (8).

#### 2.2.2 CSF Metabolites

CSF metabolite data were derived from a study by Panyard et al. (2021), which analyzed 338 metabolites in CSF samples from 291 individuals of European descent (Table S1). These metabolites included amino acids, lipids, nucleotides, and other small molecules associated with brain metabolism. Advanced mass spectrometry techniques were employed to obtain high‐resolution data on metabolites relevant to neurological disorders.

#### 2.2.3 CP Risk Data

GWAS summary statistics for CP were retrieved from the FinnGen consortium (R12 release, https://www.finngen.fi). This dataset was based on a large‐scale GWAS involving individuals of European ancestry, including 4661 CP cases and 495,687 controls (Kurki et al. 2023). Approximately 16.38 million genetic variants were analyzed under stringent quality control measures and processed using advanced imputation methods (Table S10).

### 2.3 IV Selection

For both serum and CSF metabolites, single nucleotide polymorphisms (SNPs) significantly associated with each metabolite (*p* < 1 × 10^−5^) were selected as IVs (Panyard et al. 2021; Niu et al. 2025) (Tables S2 and S4). To account for linkage disequilibrium (LD), clumping was performed using the 1000 Genomes Project European reference panel (Phase 3) with an LD threshold of *r* ^2^ < 0.001 and a physical distance window of 10,000 kb, ensuring independence among selected SNPs. The strength of each SNP as an instrument was assessed using the *F*‐statistic, with *F* > 10 indicating a strong instrument (Burgess et al. 2019). SNPs with *F*‐statistics below this threshold were excluded to prevent weak instrument bias. Potential pleiotropy was examined using MR‐Egger regression to evaluate whether genetic variants influenced CP through pathways other than metabolite alterations. For reverse MR analysis, CP GWAS data were also filtered using the same significance threshold (*p* < 1 × 10^−5^) (Table S11).

### 2.4 Statistical Analysis

#### 2.4.1 Primary MR Analysis

The inverse variance weighted (IVW) method was used as the primary MR approach, as it is the most efficient and widely applied method for estimating overall causal effects by aggregating individual SNP‐exposure and SNP‐outcome associations (Burgess et al. 2019). Under the assumption of no directional pleiotropy, the IVW method provided the most reliable causal estimates (Sanderson et al. 2022). In light of the exploratory nature of high‐throughput metabolomic analyses, a two‐stage analytical strategy was implemented:

1. **Primary analysis**: Causal associations were defined by IVW estimates passing Bonferroni correction (serum: (p < 3.6∖times 10^ (The ICGC/TCGA Pan‐Cancer Analysis of Whole Genomes Consortium 2020)); CSF: (p < 1.5∖times 10^ (The ICGC/TCGA Pan‐Cancer Analysis of Whole Genomes Consortium 2020))) or Benjamini‐Hochberg FDR correction (q < 0.05).
2. **Exploratory analysis**: If no significant associations survived multiple testing corrections, metabolites with nominal significance (p < 0.005) and concordant directional effects across MR methods (IVW/MR‐Egger (Yang et al. 2020)/weighted mode method (Sørensen et al. 2021)) were prioritized for hypothesis generation, following established practices in omics studies.

#### 2.4.2 Sensitivity Analysis

Several sensitivity analyses were conducted to assess the robustness of the MR estimates. Heterogeneity across SNPs was evaluated using Cochran's *Q* test, with *p* < 0.05 indicating significant heterogeneity (Melo et al. 2023). Directional pleiotropy was assessed using MR‐Egger regression, whereas the MR‐PRESSO method was applied to detect and correct for potential outliers (Mugat et al. 2020). Additionally, leave‐one‐out analysis was performed to determine the influence of individual SNPs on the overall causal estimate (Dahabreh 2021). The statistical software and its packages are in R (v4.3.2).

### 2.5 Metabolic Pathway Analysis

To explore metabolic pathways potentially associated with CP, pathway analysis was conducted using **MetaboAnalyst 5.0** (https://www.metaboanalyst.ca) (Pang et al. 2021). Only metabolites reaching statistical significance (*p* < 0.05) were included. The analysis incorporated two major databases: the **Small Molecule Pathway Database (SMPDB)** (Kim et al. 2020) and the **Kyoto Encyclopedia of Genes and Genomes (KEGG)** (**Kanehisa et al**. 2022). The significance threshold for pathway analysis was set at *p* < 0.05.

## 3 Results

### 3.1 MR Analysis of Serum Metabolites

**FIGURE 1:**
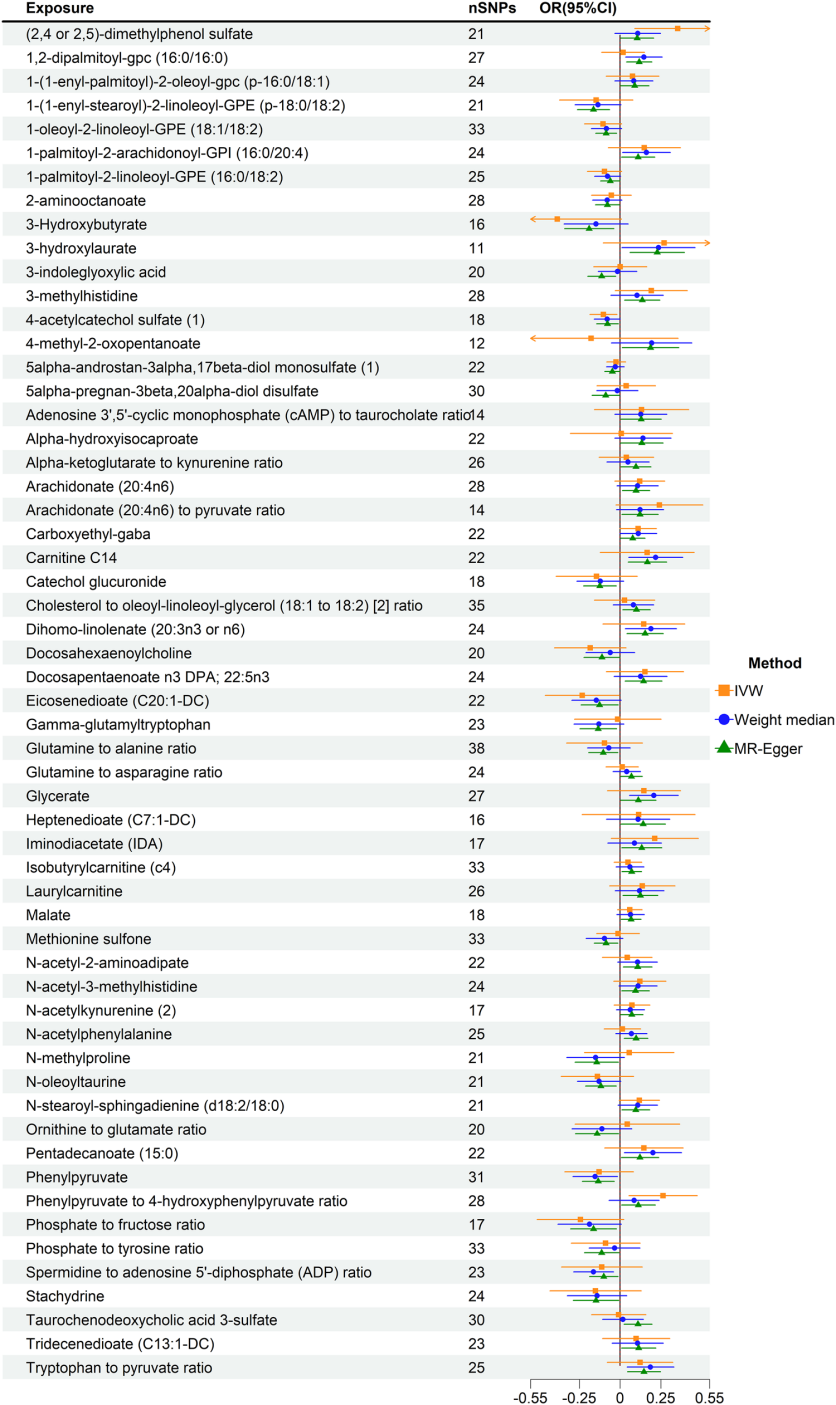
MR analysis of serum metabolites. CI, confidence interval; IVW, inverse variance weighted; MR, Mendelian randomization; OR, odd ratios.

To systematically investigate the causal relationships between serum metabolites and CP, we conducted a forward MR analysis, considering 1400 serum metabolites as exposures and CP as the outcome. A total of 69 metabolites exhibited significant associations with CP risk (*p* < 0.05) (Table S3). Among these, 13 were unidentified compounds, highlighting the need for further structural elucidation. After stringent Bonferroni correction, no serum or CSF metabolites reached statistical significance in the primary MR analysis. However, in exploratory analyses using a relaxed threshold (*p* < 0.005), three serum metabolites, 1‐(1‐enyl‐stearoyl)‐2‐linoleoyl‐GPE (IVW: odd ratio [OR] = 0.84, 95% confidence interval [CI]: 0.77–0.94, *p* = 0.001), 1,2‐dipalmitoyl‐GPC (IVW: OR = 1.12, 95% CI: 1.04–1.21, *p* = 0.003), and the tryptophan‐to‐pyruvate ratio (IVW: OR = 1.15, 95% CI: 1.04–1.28, *p* = 0.005), demonstrated nominally significant associations with CP risk (Figure 1; Figures S1–S8). These findings suggest that alterations in lipid metabolism and amino acid pathways may play a role in CP pathophysiology.

### 3.2 MR Analysis of CSF Metabolites

**FIGURE 2:**
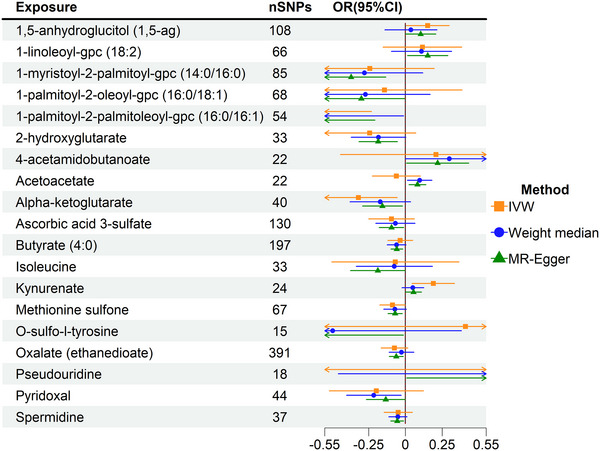
MR analysis of CSF metabolites. CI, confidence interval; IVW, inverse variance weighted; MR, Mendelian randomization; OR, odd ratios.

Given the critical role of CSF in reflecting central nervous system metabolic alterations, we extended our MR analysis to 338 CSF metabolites. A total of 13 metabolites exhibited significant associations with CP risk (*p* < 0.05) (Table S5). Among these, three remained unidentified, warranting further investigation. After stringent Bonferroni correction, no serum or CSF metabolites reached statistical significance in the primary MR analysis. However, in exploratory analyses using a relaxed threshold (*p* < 0.005), three CSF metabolites, 1‐palmitoyl‐2‐palmitoleoyl‐GPC (16:0/16:1) (IVW: OR = 0.57, 95% CI: 0.40–0.81, *p* = 0.001), 1‐myristoyl‐2‐palmitoyl‐GPC (14:0/16:0) (IVW: OR = 0.69, 95% CI: 0.54–0.87, *p* = 0.002), and butyrate (4:0) (IVW: OR = 0.94, 95% CI: 0.90–0.98, *p* = 0.004) (Figure 2; Figures S9‐S12). These results indicate potential neuroprotective roles of specific glycerophospholipids and short‐chain fatty acids in CP.

### 3.3 Shared Significant Metabolites in CSF and Serum

**FIGURE 3:**
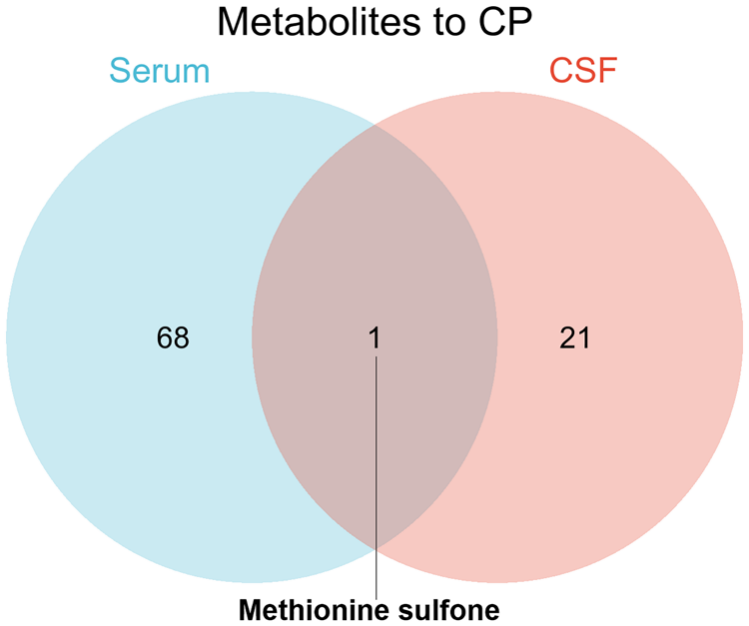
Shared significant metabolites in CSF and serum. CP, cerebral palsy; CSF, cerebrospinal fluid.

**FIGURE 4:**
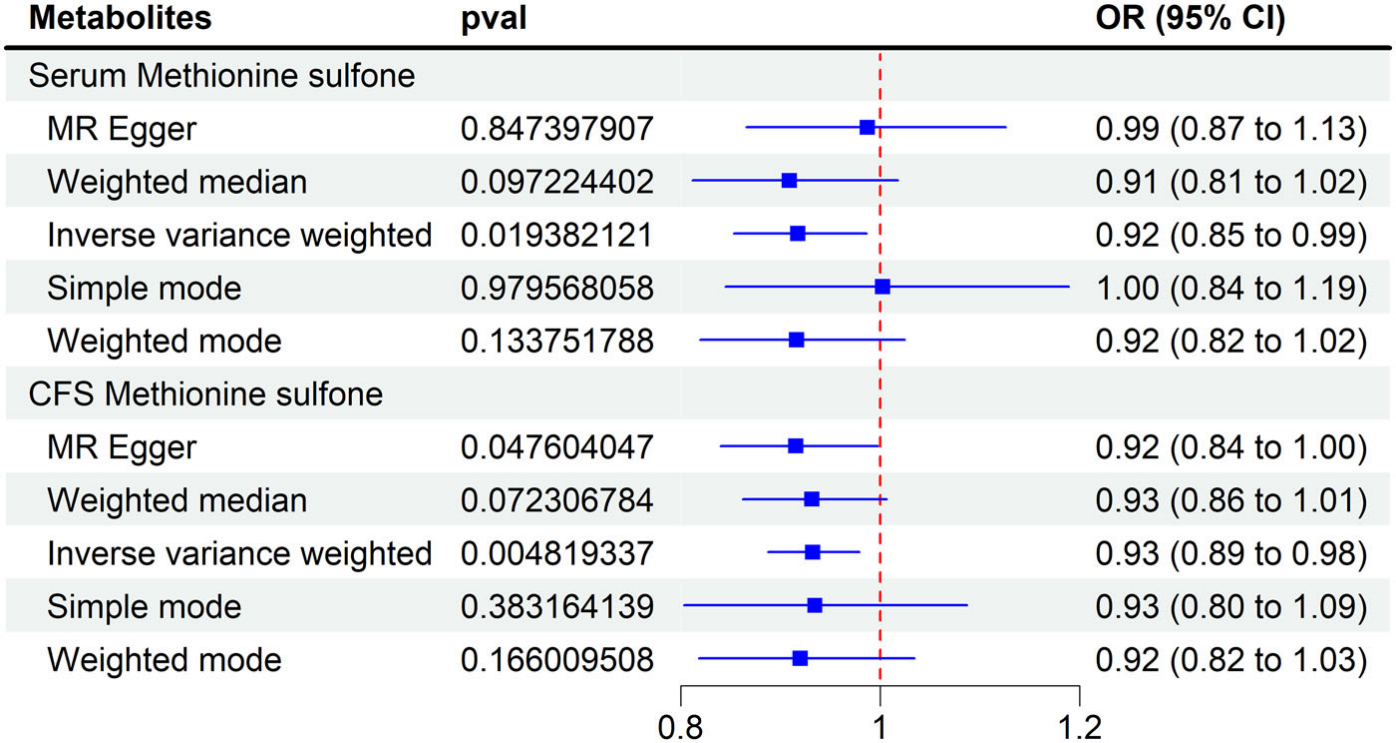
MR analysis of methionine sulfone in serum and CSF metabolites. CI, confidence interval; CSF, cerebrospinal fluid; MR, Mendelian randomization; OR, odd ratios.

To identify common metabolic signatures between CSF and serum, we performed an integrative analysis, revealing that methionine sulfone was significantly associated with CP in both biofluids (serum: OR = 0.91, 95% CI: 0.85–0.99, *p* = 0.02; reverse OR = 0.93, 95% CI: 0.89–1.09, *p* = 0.04) (Figure 4). The consistent protective effect observed in both compartments suggests that methionine sulfone may serve as a promising biomarker for CP, warranting further functional validation (Duan et al. 2024; Wang et al. 2024) (Figure 3 ; Table S7).

### 3.4 Metabolic Pathway Analysis

To gain mechanistic insights into CP‐related metabolic alterations, we performed pathway enrichment analysis using Metabolic Analyzer 5.0. The results indicated that glyoxylate and dicarboxylate metabolism, biosynthesis of unsaturated fatty acids, and glycerophospholipid metabolism were significantly enriched in serum metabolites. In contrast, the butyrate metabolism pathway (*p* = 1.04E − 06, FDR = 8.32E − 05) was highly enriched in CSF, with four metabolites (acetoacetate, butanoic acid, 2‐oxoglutarate, and 2‐hydroxyglutarate) participating in this pathway (Tables S2 and S9). These findings underscore the role of fatty acid metabolism in CP pathogenesis and highlight potential therapeutic targets.

### 3.5 Sensitivity and Heterogeneity Analyses

**FIGURE 5:**
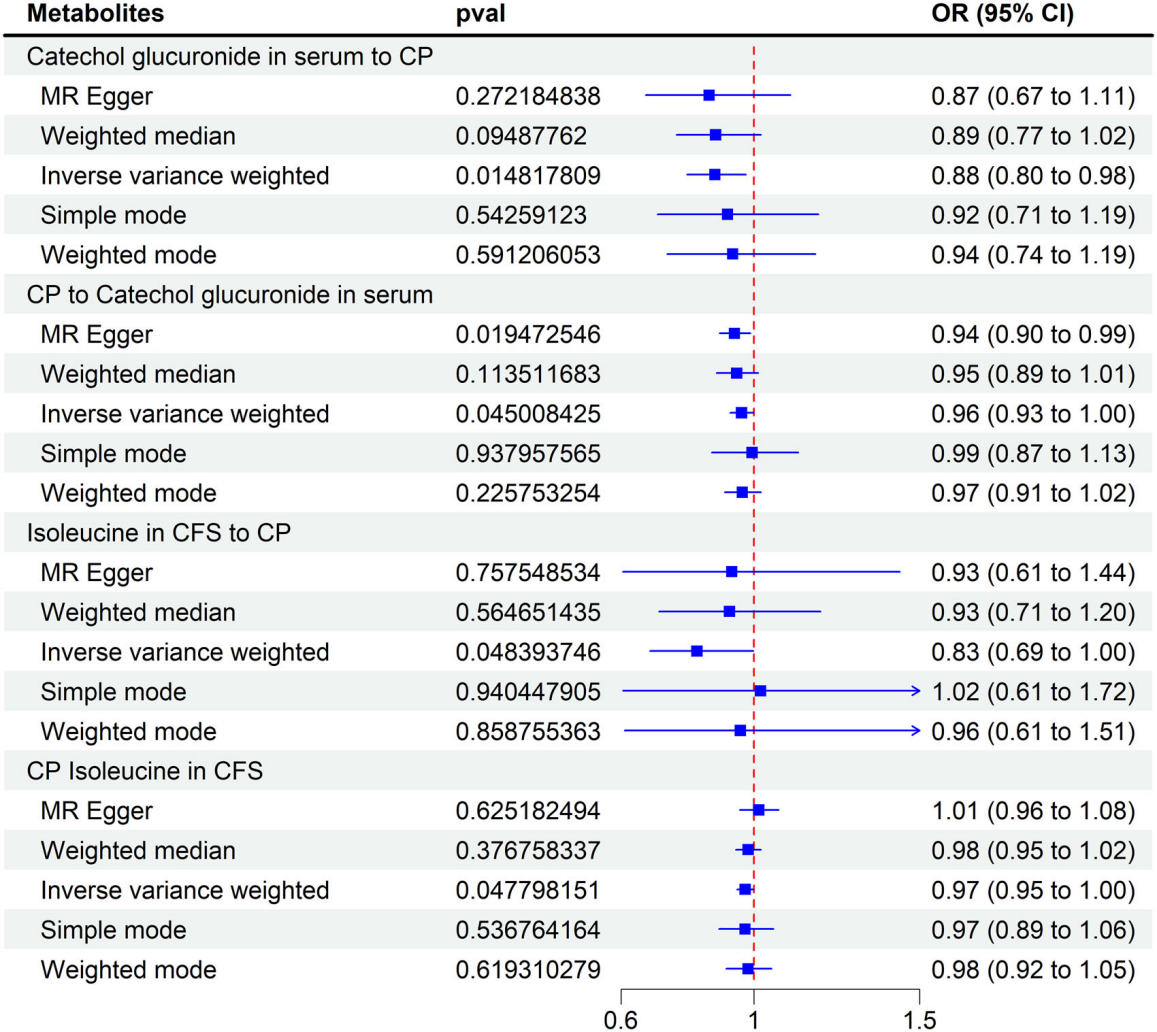
Reverse MR Analysis of catechol glucuronide in serum and isoleucine in CSF. CI, confidence interval; CP, cerebral palsy; CSF, cerebrospinal fluid; MR, Mendelian randomization; OR, odd ratios.

To ensure the robustness of our MR estimates, we performed multiple sensitivity analyses. Cochran's *Q*‐test indicated no significant heterogeneity among SNPs (*p* > 0.05), suggesting the validity of our IVs. Moreover, the MR‐Egger intercept test provided no evidence of directional pleiotropy (*p* > 0.05), and the MR‐PRESSO method did not detect any influential outliers. Leave‐one‐out analyses further confirmed that no single SNP disproportionately affected the causal estimates, reinforcing the reliability of our findings (Figure 5; Table S6).

### 3.6 Reverse MR Analysis

To assess the bidirectionality of metabolic alterations in CP, we performed a reverse MR analysis, using CP as the exposure and previously identified metabolites in serum and CSF as outcomes. IVW analysis revealed significant bidirectional relationships for catechol glucuronide in serum (forward: OR = 0.96, 95% CI: 0.92–0.99, *p* = 0.01; reverse OR = 1.36, 95% CI: 1.08–1.71, *p* = 0.04) and isoleucine in CSF (forward: OR = 0.82, 95% CI: 0.69–0.99, *p* = 0.04; reverse OR = 0.97, 95% CI: 0.94–0.99, *p* = 0.04). Both metabolites exhibited reduced concentrations in CP cases (Table S8), further supporting their potential involvement in CP pathophysiology. Future studies are warranted to elucidate their precise mechanistic roles in CP development (Figure 3).

## 4 Discussion

Despite advances in clinical characterization and neuroanatomical studies, the molecular mechanisms underlying CP remain incompletely understood, posing challenges for early diagnosis and the identification of effective therapeutic targets. Therefore, elucidating the pathophysiological mechanisms of CP and identifying potential biomarkers are of paramount importance (Chen et al. 2021). Current clinical guidelines for CP diagnosis, such as the Hammersmith Infant Neurological Examination (HINE) and brain MRI protocols endorsed by international consortia, achieve 85%–92% sensitivity in high‐risk infants but lack biomarkers for preclinical detection or subtype stratification (Novak et al. 2017; Bax et al. 2005; Hadders‐Algra et al. 2017). In this study, a two‐sample MR approach was employed to assess the causal effects of serum and CSF metabolites on CP. Although no metabolites survived rigorous multiple testing correction (Bonferroni: serum *p* < 3.6 × 10^−5^, CSF *p* < 1.5 × 10^−4^), exploratory analyses identified 12 serum metabolites (e.g., 1‐(1‐enyl‐stearoyl)‐2‐linoleoyl‐GPE, *p* = 0.0012) and 3 CSF metabolites (e.g., 1‐palmitoyl‐2‐palmitoleoyl‐GPC, *p* = 0.003) with nominally significant associations and consistent directional effects across MR methods. These findings align with prior studies linking glycerophospholipid metabolism to neurodevelopmental disorders, suggesting potential biological relevance despite the need for cautious interpretation (Varga et al. 2024). Notably, the protective metabolite methionine sulfone and dysregulated glyoxylate/dicarboxylate pathway identified here could enhance existing diagnostic frameworks. For instance, integrating methionine sulfone levels with HINE scores may improve early detection specificity in infants with equivocal neuroimaging findings, whereas pathway analysis provides a molecular basis for subclassifying CP phenotypes (Hadders‐Algra et al. 2017). These results not only address a critical knowledge gap but also lay the foundation for future research and clinical applications, underscoring the significance of metabolomics in the study of neurological disorders (Yan et al. 2022).

Through MR analysis, a significant causal relationship between specific metabolites in serum and CSF and CP was identified. Notably, 69 serum metabolites were significantly associated with CP risk, including **1‐(1‐enyl‐stearoyl)‐2‐linoleoyl‐GPE** and **1,2‐dipalmitoyl‐GPC**. These findings suggest a potential role for metabolites in CP pathogenesis and offer new directions for biomarker research. The biological relevance of these results lies in the possibility that these metabolites reflect biochemical alterations related to neuronal damage. Existing literature supports the association between serum metabolite alterations and motor dysfunction, indicating that these metabolites may serve as potential targets for early intervention and treatment strategies (Chen et al. 2021; Hanaoka et al. 2024).

In the analysis of CSF metabolites, 13 metabolites were significantly associated with CP risk, with **1‐palmitoyl‐2‐palmitoleoyl‐GPC** exhibiting the most pronounced protective effect. This finding provides novel insights into the neurobiological mechanisms of CP and underscores the importance of CSF in neurological disease research. Previous studies have suggested that CSF metabolite alterations are closely linked to the extent of neuronal damage and its pathological progression. Future research should explore whether these metabolites could serve as potential biomarkers for assessing disease severity or monitoring treatment efficacy (Varga et al. 2024; Yan et al. 2022).

Additionally, a shared significant metabolite, **methionine sulfone**, was identified in both serum and CSF, demonstrating a protective effect in both biofluids. This finding highlights the consistency of metabolic alterations across different biofluids and suggests its biological significance in CP. The literature indicates that shared metabolite research can contribute to biomarker development for other neurological disorders. Future studies should focus on translating these metabolic findings into clinical practice to facilitate more precise therapeutic strategies for CP patients (Yan et al. 2023, 2022).

Furthermore, metabolic pathway analysis revealed that specific pathways, particularly **glyoxylate metabolism and butyrate metabolism**, played a crucial role in CP pathogenesis, with butyrate metabolism being particularly noteworthy. These findings provide new perspectives on the pathophysiological mechanisms of CP and offer potential targets for therapeutic intervention. Previous studies have demonstrated that metabolic pathway regulation can influence neuroprotective mechanisms, further promoting the development of personalized medicine. Future research should investigate whether modulating these pathways could offer novel therapeutic strategies for CP patients (Álvarez Zaragoza et al. 2019).

This study has certain limitations. The lack of experimental validation limits the confirmation of the biological mechanisms underlying the findings. Additionally, the sample size may constrain the generalizability of the results, particularly across different populations and regions. Moreover, integrating multiple datasets may introduce batch effects, potentially affecting result reliability. Future studies should incorporate larger and more diverse cohorts while strengthening experimental validation to further elucidate the role of metabolites in CP.

## 5 Conclusion

This study identified potential causal relationships between serum and CSF metabolites and CP, providing a crucial foundation for future biomarker research and clinical applications. By applying a two‐sample MR approach, specific metabolites were identified as significantly associated with CP risk, suggesting their potential involvement in disease mechanisms. Future research should further explore the biological significance of these metabolites and their feasibility as early diagnostic tools, ultimately advancing early intervention and personalized treatment for CP.

## Author Contributions

Yonggang Dai conceived the study, conducted formal analyses and investigations, developed the methodology, validated findings, and contributed to manuscript revisions. Hongya Wang managed data curation, performed formal analyses, coordinated project administration, provided resources, created visualizations, and participated in critical editing. Xuewei Zhuang engaged in formal analysis, experimental investigations and validation processes and contributed to both original drafting and manuscript refinement. Wei Wang secured funding, oversaw project administration and supervision, and participated in initial manuscript drafting. All authors reviewed and approved the final version.

## Disclosure

This study used publicly available summary data from previously published GWAS.

## Ethics Statement

The authors have nothing to report.

## Consent

The authors have nothing to report.

## Conflicts of Interest

The authors declare no conflicts of interest.

## Peer Review

The peer review history for this article is available at https://publons.com/publon/10.1002/brb3.70864.

## Supporting information



Supplementary Material: brb370864‐sup‐0001‐FigureS1‐S12.docx

Supplementary Material: brb370864‐sup‐0002‐TableS1‐S11.xlsx

## Data Availability

All original GWAS had obtained informed consent from participants, and the data were de‐identified and publicly available for research purposes.
