# Supplementary material for: Evaluating the Causal Effects of Serum and Cerebrospinal Fluid Metabolites on Cerebral Palsy: A Whole‐Metabolome Mendelian Randomization Study
Source: Brain Behav. 2025 Sep 21;15(9):e70864. doi: 10.1002/brb3.70864 (PMC12451031; doi:10.1002/brb3.70864)
Supplement: Supplementary file 1 — Supplementary Material: brb370864‐sup‐0001‐FigureS1‐S12.docx [file BRB3-15-e70864-s001.docx]

**Supplementary Figures**

**Figure legends**

Fig. S1: Forest plot for MR result of the serum metabolites and CP No1.

Fig. S2: Forest plot for MR result of the serum metabolites and CP No2.

Fig. S3: Funnel plot for MR result of the serum metabolites and CP No1.

Fig. S4: Funnel plot for MR result of the serum metabolites and CP No2.

Fig. S5: Scatter plot for MR result of the serum metabolites and CP No1.

Fig. S6: Scatter plot for MR result of the serum metabolites and CP No2.

Fig. S7: Leave-one-out plot for MR result of the serum metabolic and CP No1.

Fig. S8: Leave-one-out plot for MR result of the serum metabolic and CP No2.

Fig. S9: Forest plot for MR result of CSF metabolites and CP;

Fig. S10: Funnel plot for MR result of CSF metabolites and CP;

Fig. S11: Scatter plot for MR result of CSF metabolites and CP;

Fig. S12: Leave one out for MR result of CSF metabolites and CP;


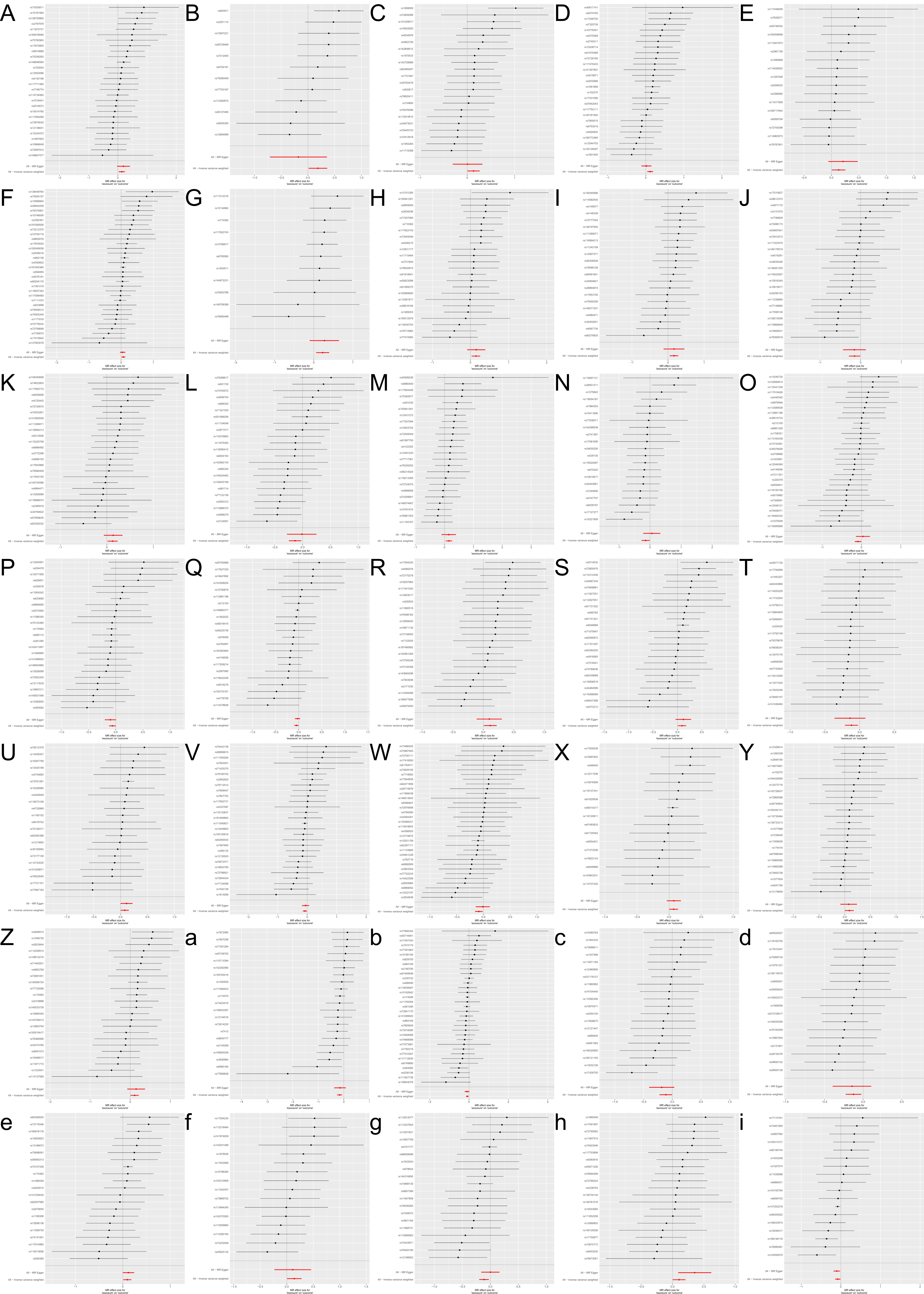


**Fig. S1: Forest plot for MR result of the serum metabolites and CP No1.**

(A)3-methylhistidine; (B)4-methyl-2-oxopentanoate; (C)Alpha-hydroxyisocaproate; (D)1,2-dipalmitoyl-gpc (16:0/16:0); (E)Iminodiacetate (IDA); (F)Isobutyrylcarnitine (c4); (G)3-hydroxylaurate; (H)Docosapentaenoate n3 DPA; 22:5n3; (I)Carnitine C14; (J)Stachydrine; (K)Laurylcarnitine; (L)Gamma-glutamyltryptophan; (M)Dihomo-linolenate (20:3n3 or n6); (N)N-methylproline; (O)5alpha-pregnan-3beta,20alpha-diol disulfate; (P)1-palmitoyl-2-linoleoyl-GPE (16:0/18:2); (Q)5alpha-androstan-3alpha,17beta-diol monosulfate (1); (R)Tridecenedioate (C13:1-DC); (S)N-acetyl-3-methylhistidine; (T)N-oleoyltaurine; (U)Carboxyethyl-gaba; (V)2-aminooctanoate; (W)Methionine sulfone; (X)N-acetylkynurenine (2); (Y)1-(1-enyl-palmitoyl)-2-oleoyl-gpc (p-16:0/18:1); (Z)1-palmitoyl-2-arachidonoyl-GPI (16:0/20:4); (a)1-(1-enyl-stearoyl)-2-linoleoyl-GPE (p-18:0/18:2); (b)1-oleoyl-2-linoleoyl-GPE (18:1/18:2); (c)Docosahexaenoylcholine; (d)Catechol glucuronide; (e)N-stearoyl-sphingadienine (d18:2/18:0); (f)Heptenedioate (C7:1-DC); (g)3-indoleglyoxylic acid; (h)(2,4 or 2,5)-dimethylphenol sulfate; (i)4-acetylcatechol sulfate (1)

**
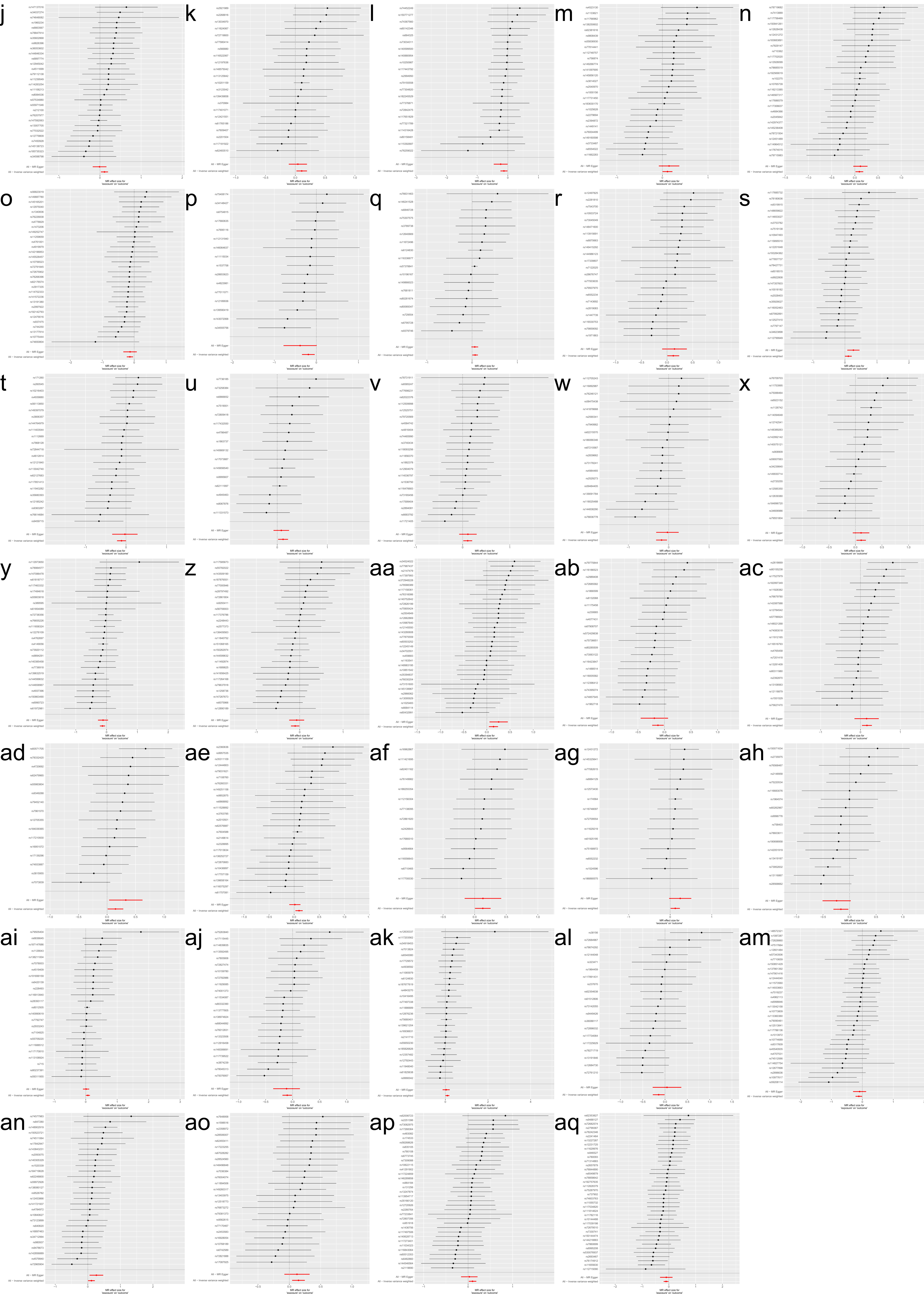
**

**Fig. S2: Forest plot for MR result of the serum metabolites and CP No2.**

(j)Taurochenodeoxycholic acid 3-sulfate; (k)N-acetyl-2-aminoadipate; (l)Eicosenedioate (C20:1-DC); (m)Glycerate; (n)Arachidonate (20:4n6); (o)Phenylpyruvate; (p)3-Hydroxybutyrate; (q)Malate; (r)Pentadecanoate (15:0); (s)X-11483; (t)X-12216; (u)X-12411; (v)X-12013; (w)X-13728; (x)X-14939; (y)X-17653; (z)X-21258; (a)X-21736; (b)X-22834; (c)X-25343; (d)X-25520; (e)N-acetylphenylalanine; (f)Adenosine 3',5'-cyclic monophosphate (cAMP) to taurocholate ratio; (g)Arachidonate (20:4n6) to pyruvate ratio; (h)Phosphate to fructose ratio; (i)Glutamine to asparagine ratio; (j)Spermidine to adenosine 5'-diphosphate (ADP) ratio; (k)Alpha-ketoglutarate to kynurenine ratio; (l)Ornithine to glutamate ratio; (m)Phosphate to tyrosine ratio; (n)Phenylpyruvate to 4-hydroxyphenylpyruvate ratio; (o)Tryptophan to pyruvate ratio; (p)Cholesterol to oleoyl-linoleoyl-glycerol (18:1 to 18:2) [2] ratio; (q)Glutamine to alanine ratio


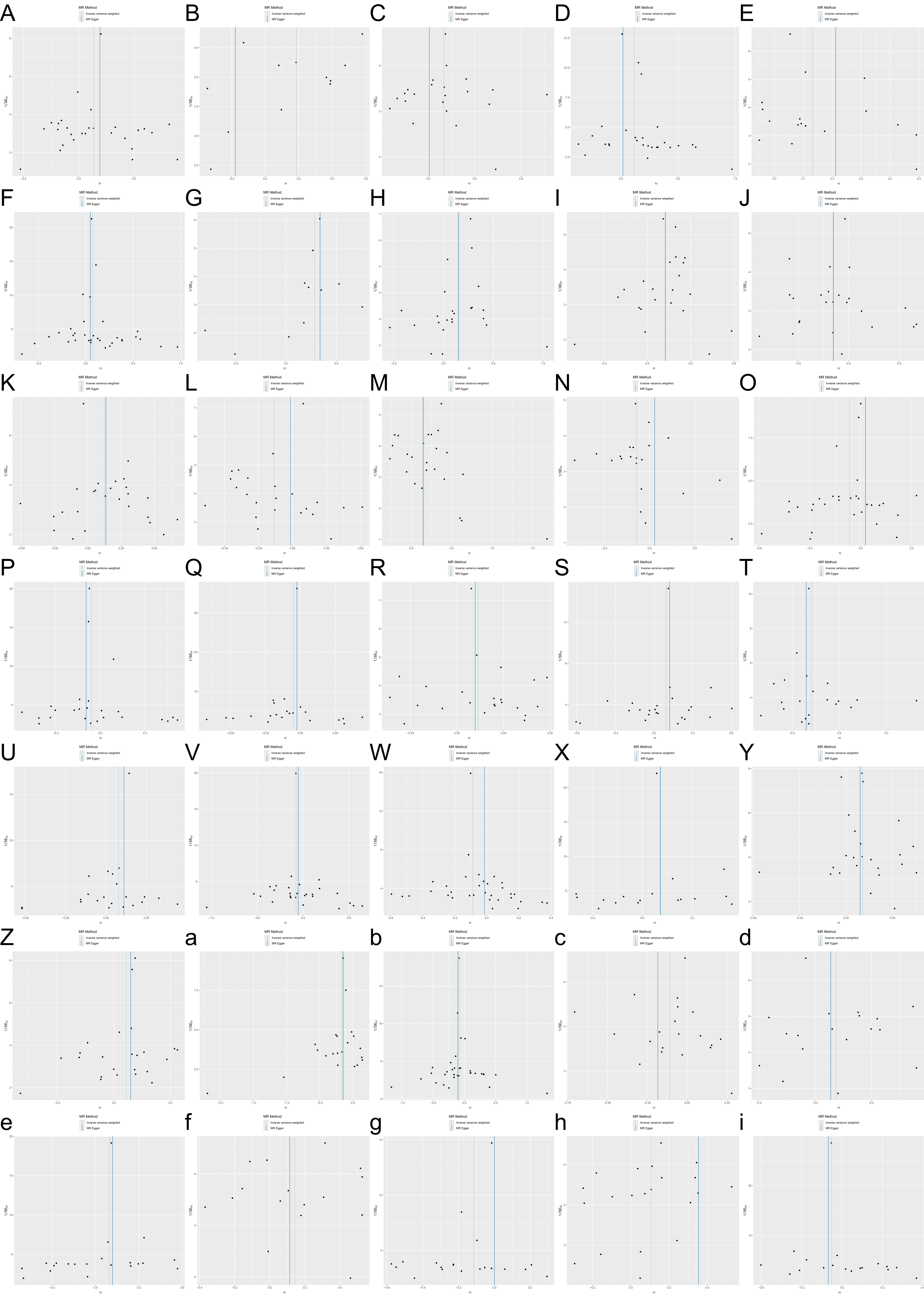


**Fig. S3: Funnel plot for MR result of the serum metabolites and CP No1.**

(A)3-methylhistidine; (B)4-methyl-2-oxopentanoate; (C)Alpha-hydroxyisocaproate; (D)1,2-dipalmitoyl-gpc (16:0/16:0); (E)Iminodiacetate (IDA); (F)Isobutyrylcarnitine (c4); (G)3-hydroxylaurate; (H)Docosapentaenoate n3 DPA; 22:5n3; (I)Carnitine C14; (J)Stachydrine; (K)Laurylcarnitine; (L)Gamma-glutamyltryptophan; (M)Dihomo-linolenate (20:3n3 or n6); (N)N-methylproline; (O)5alpha-pregnan-3beta,20alpha-diol disulfate; (P)1-palmitoyl-2-linoleoyl-GPE (16:0/18:2); (Q)5alpha-androstan-3alpha,17beta-diol monosulfate (1); (R)Tridecenedioate (C13:1-DC); (S)N-acetyl-3-methylhistidine; (T)N-oleoyltaurine; (U)Carboxyethyl-gaba; (V)2-aminooctanoate; (W)Methionine sulfone; (X)N-acetylkynurenine (2); (Y)1-(1-enyl-palmitoyl)-2-oleoyl-gpc (p-16:0/18:1); (Z)1-palmitoyl-2-arachidonoyl-GPI (16:0/20:4); (a)1-(1-enyl-stearoyl)-2-linoleoyl-GPE (p-18:0/18:2); (b)1-oleoyl-2-linoleoyl-GPE (18:1/18:2); (c)Docosahexaenoylcholine; (d)Catechol glucuronide; (e)N-stearoyl-sphingadienine (d18:2/18:0); (f)Heptenedioate (C7:1-DC); (g)3-indoleglyoxylic acid; (h)(2,4 or 2,5)-dimethylphenol sulfate; (i)4-acetylcatechol sulfate (1)


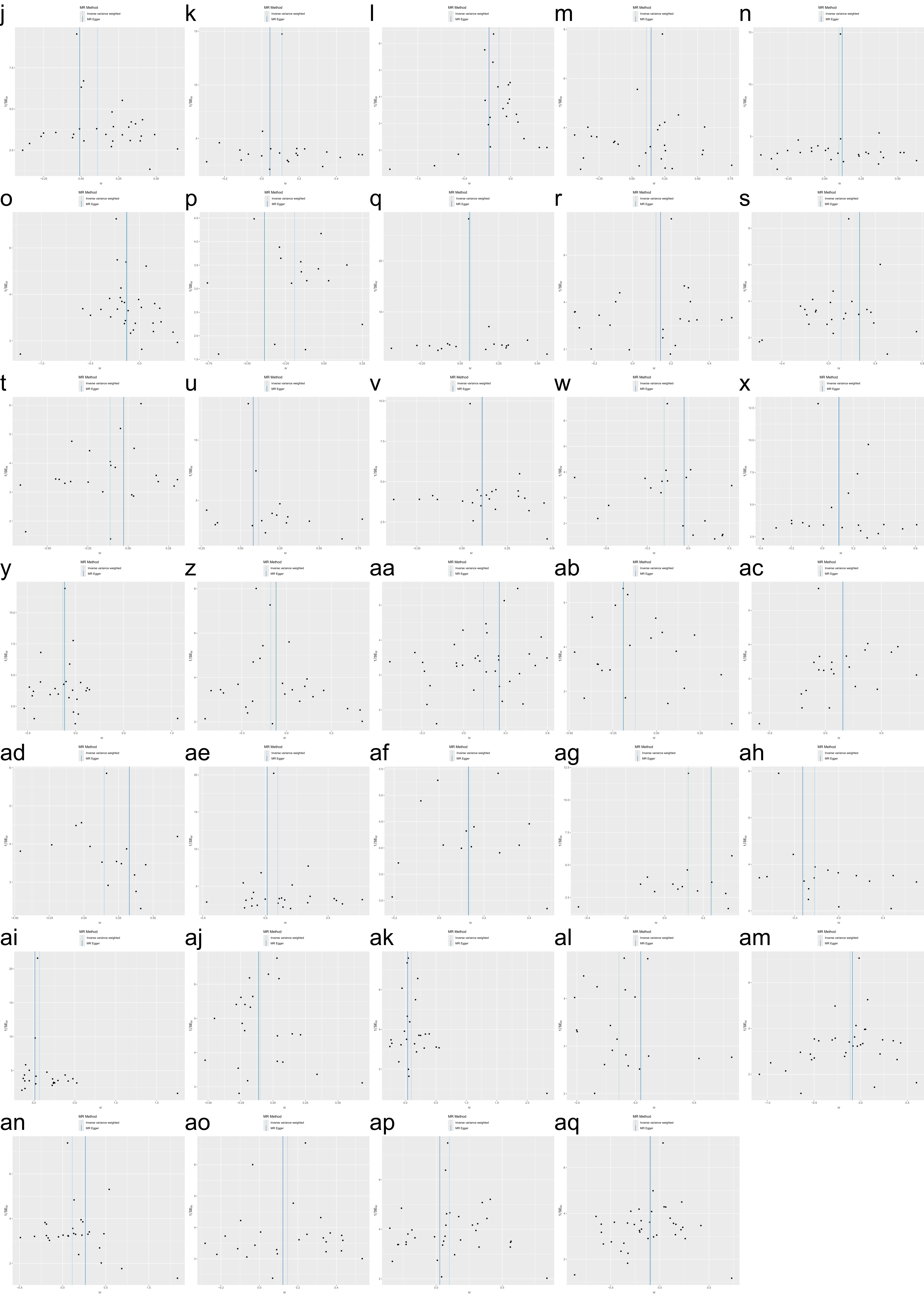


**Fig. S4: Funnel plot for MR result of the serum metabolites and CP No2.**

(j)Taurochenodeoxycholic acid 3-sulfate; (k)N-acetyl-2-aminoadipate; (l)Eicosenedioate (C20:1-DC); (m)Glycerate; (n)Arachidonate (20:4n6); (o)Phenylpyruvate; (p)3-Hydroxybutyrate; (q)Malate; (r)Pentadecanoate (15:0); (s)X-11483; (t)X-12216; (u)X-12411; (v)X-12013; (w)X-13728; (x)X-14939; (y)X-17653; (z)X-21258; (a)X-21736; (b)X-22834; (c)X-25343; (d)X-25520; (e)N-acetylphenylalanine; (f)Adenosine 3',5'-cyclic monophosphate (cAMP) to taurocholate ratio; (g)Arachidonate (20:4n6) to pyruvate ratio; (h)Phosphate to fructose ratio; (i)Glutamine to asparagine ratio; (j)Spermidine to adenosine 5'-diphosphate (ADP) ratio; (k)Alpha-ketoglutarate to kynurenine ratio; (l)Ornithine to glutamate ratio; (m)Phosphate to tyrosine ratio; (n)Phenylpyruvate to 4-hydroxyphenylpyruvate ratio; (o)Tryptophan to pyruvate ratio; (p)Cholesterol to oleoyl-linoleoyl-glycerol (18:1 to 18:2) [2] ratio; (q)Glutamine to alanine ratio


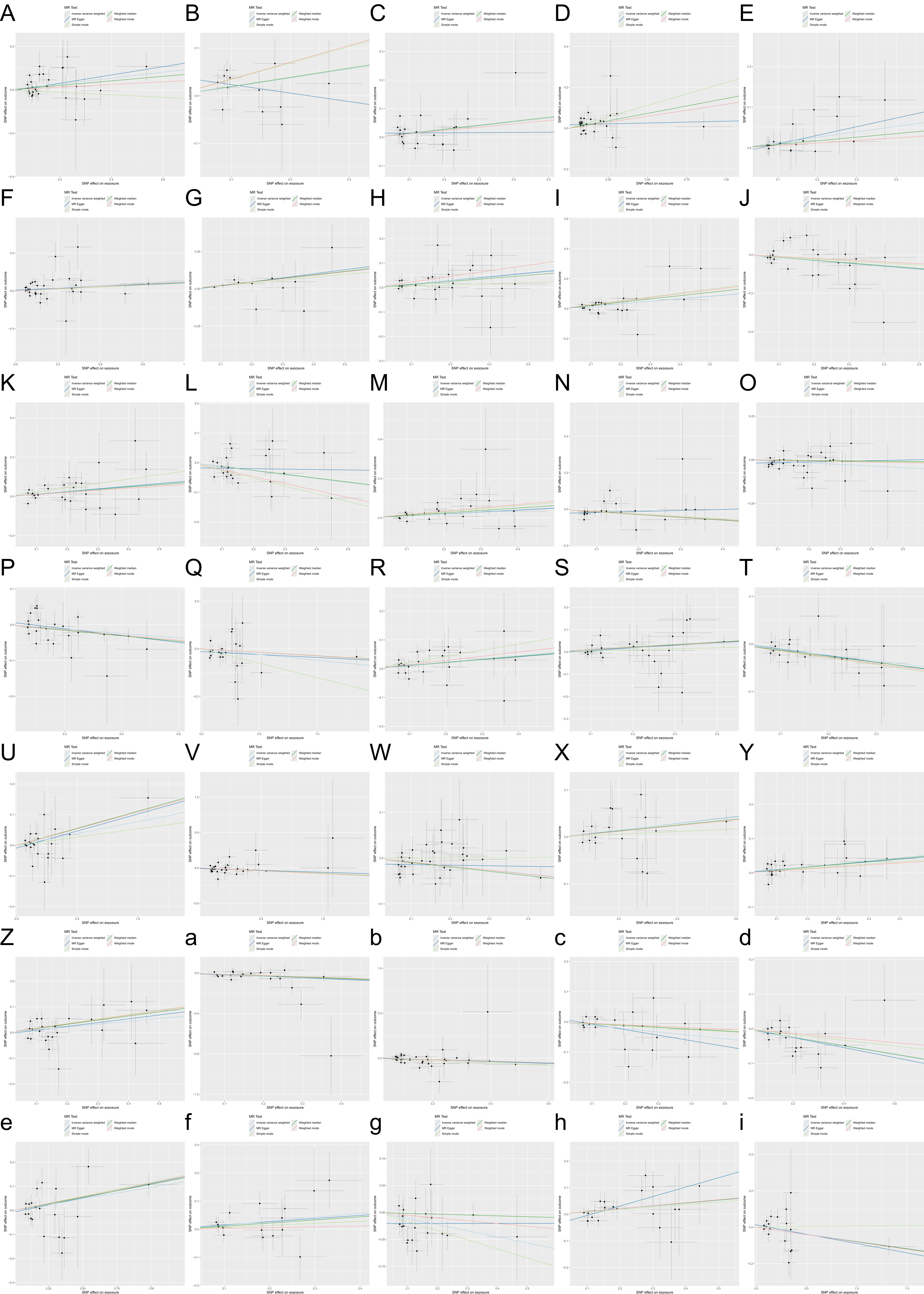


**Fig. S5: Scatter plot for MR result of the serum metabolites and CP No1**.

(A)3-methylhistidine; (B)4-methyl-2-oxopentanoate; (C)Alpha-hydroxyisocaproate; (D)1,2-dipalmitoyl-gpc (16:0/16:0); (E)Iminodiacetate (IDA); (F)Isobutyrylcarnitine (c4); (G)3-hydroxylaurate; (H)Docosapentaenoate n3 DPA; 22:5n3; (I)Carnitine C14; (J)Stachydrine; (K)Laurylcarnitine; (L)Gamma-glutamyltryptophan; (M)Dihomo-linolenate (20:3n3 or n6); (N)N-methylproline; (O)5alpha-pregnan-3beta,20alpha-diol disulfate; (P)1-palmitoyl-2-linoleoyl-GPE (16:0/18:2); (Q)5alpha-androstan-3alpha,17beta-diol monosulfate (1); (R)Tridecenedioate (C13:1-DC); (S)N-acetyl-3-methylhistidine; (T)N-oleoyltaurine; (U)Carboxyethyl-gaba; (V)2-aminooctanoate; (W)Methionine sulfone; (X)N-acetylkynurenine (2); (Y)1-(1-enyl-palmitoyl)-2-oleoyl-gpc (p-16:0/18:1); (Z)1-palmitoyl-2-arachidonoyl-GPI (16:0/20:4); (a)1-(1-enyl-stearoyl)-2-linoleoyl-GPE (p-18:0/18:2); (b)1-oleoyl-2-linoleoyl-GPE (18:1/18:2); (c)Docosahexaenoylcholine; (d)Catechol glucuronide; (e)N-stearoyl-sphingadienine (d18:2/18:0); (f)Heptenedioate (C7:1-DC); (g)3-indoleglyoxylic acid; (h)(2,4 or 2,5)-dimethylphenol sulfate; (i)4-acetylcatechol sulfate (1)


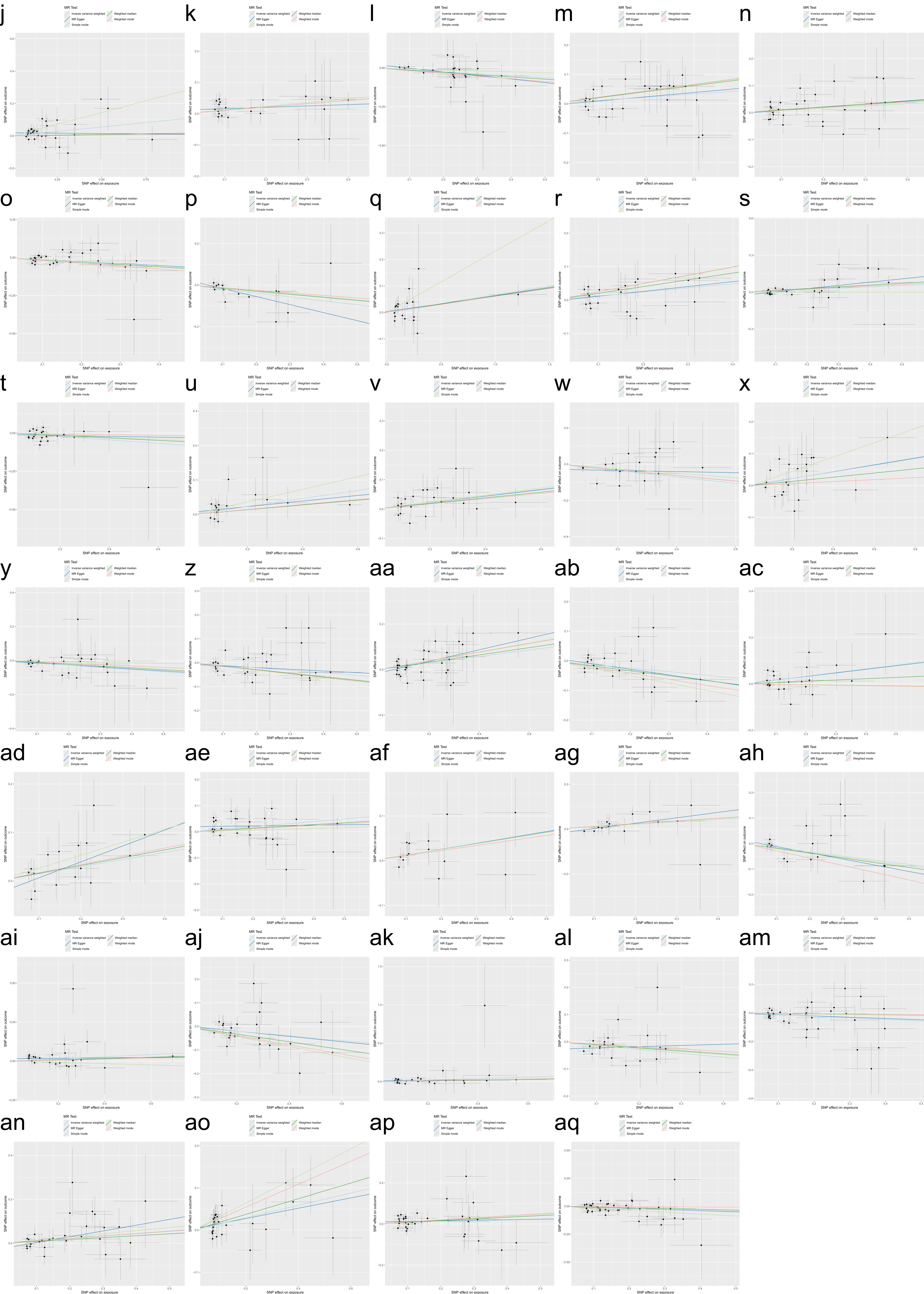


**Fig. S6: Scatter plot for MR result of the serum metabolites and CP No2.**

(j)Taurochenodeoxycholic acid 3-sulfate; (k)N-acetyl-2-aminoadipate; (l)Eicosenedioate (C20:1-DC); (m)Glycerate; (n)Arachidonate (20:4n6); (o)Phenylpyruvate; (p)3-Hydroxybutyrate; (q)Malate; (r)Pentadecanoate (15:0); (s)X-11483; (t)X-12216; (u)X-12411; (v)X-12013; (w)X-13728; (x)X-14939; (y)X-17653; (z)X-21258; (a)X-21736; (b)X-22834; (c)X-25343; (d)X-25520; (e)N-acetylphenylalanine; (f)Adenosine 3',5'-cyclic monophosphate (cAMP) to taurocholate ratio; (g)Arachidonate (20:4n6) to pyruvate ratio; (h)Phosphate to fructose ratio; (i)Glutamine to asparagine ratio; (j)Spermidine to adenosine 5'-diphosphate (ADP) ratio; (k)Alpha-ketoglutarate to kynurenine ratio; (l)Ornithine to glutamate ratio; (m)Phosphate to tyrosine ratio; (n)Phenylpyruvate to 4-hydroxyphenylpyruvate ratio; (o)Tryptophan to pyruvate ratio; (p)Cholesterol to oleoyl-linoleoyl-glycerol (18:1 to 18:2) [2] ratio; (q)Glutamine to alanine ratio
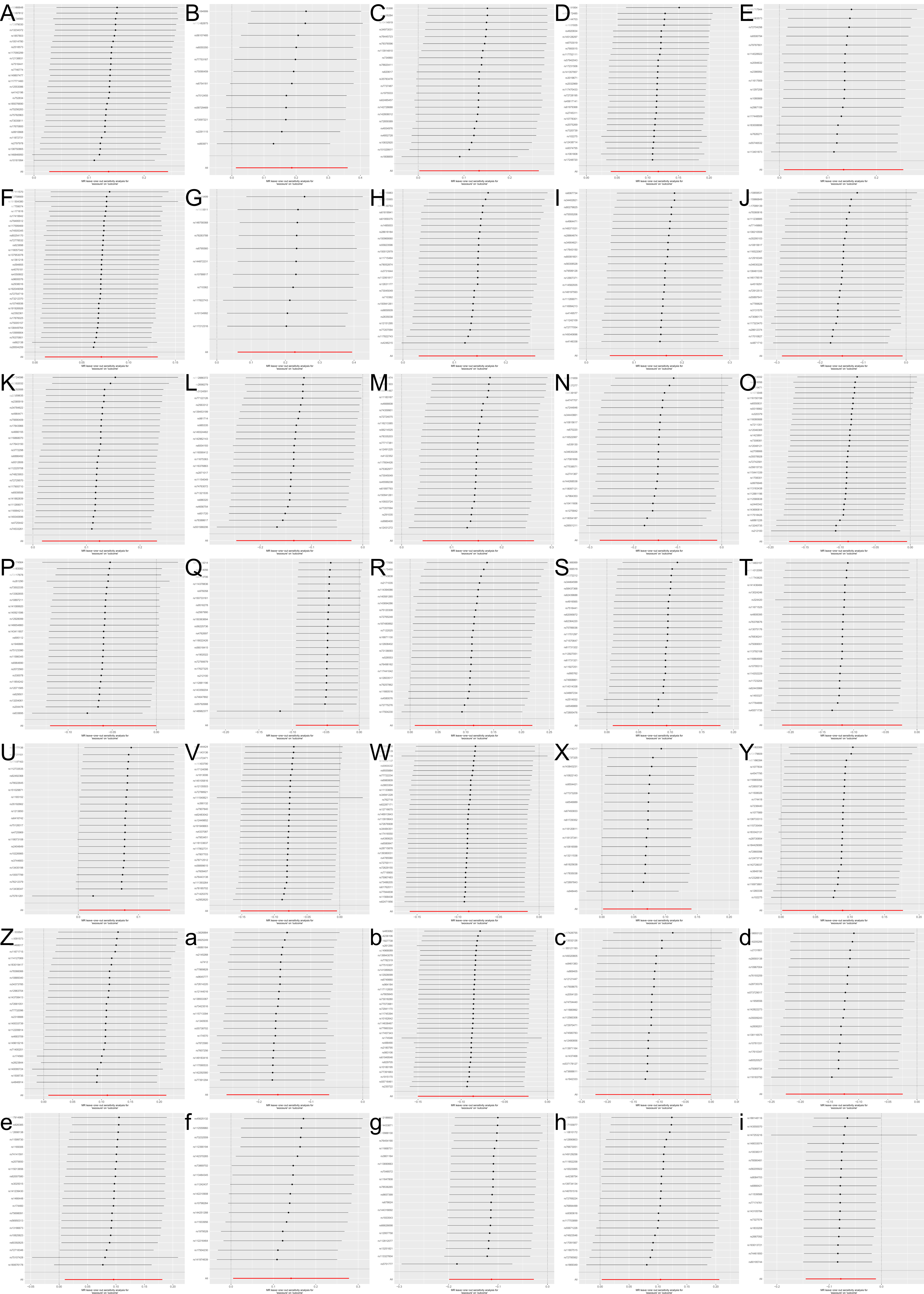


**Fig. S7: Leave-one-out plot for MR result of the serum metabolic and CP No1.**

(A)3-methylhistidine; (B)4-methyl-2-oxopentanoate; (C)Alpha-hydroxyisocaproate; (D)1,2-dipalmitoyl-gpc (16:0/16:0); (E)Iminodiacetate (IDA); (F)Isobutyrylcarnitine (c4); (G)3-hydroxylaurate; (H)Docosapentaenoate n3 DPA; 22:5n3; (I)Carnitine C14; (J)Stachydrine; (K)Laurylcarnitine; (L)Gamma-glutamyltryptophan; (M)Dihomo-linolenate (20:3n3 or n6); (N)N-methylproline; (O)5alpha-pregnan-3beta,20alpha-diol disulfate; (P)1-palmitoyl-2-linoleoyl-GPE (16:0/18:2); (Q)5alpha-androstan-3alpha,17beta-diol monosulfate (1); (R)Tridecenedioate (C13:1-DC); (S)N-acetyl-3-methylhistidine; (T)N-oleoyltaurine; (U)Carboxyethyl-gaba; (V)2-aminooctanoate; (W)Methionine sulfone; (X)N-acetylkynurenine (2); (Y)1-(1-enyl-palmitoyl)-2-oleoyl-gpc (p-16:0/18:1); (Z)1-palmitoyl-2-arachidonoyl-GPI (16:0/20:4); (a)1-(1-enyl-stearoyl)-2-linoleoyl-GPE (p-18:0/18:2); (b)1-oleoyl-2-linoleoyl-GPE (18:1/18:2); (c)Docosahexaenoylcholine; (d)Catechol glucuronide; (e)N-stearoyl-sphingadienine (d18:2/18:0); (f)Heptenedioate (C7:1-DC); (g)3-indoleglyoxylic acid; (h)(2,4 or 2,5)-dimethylphenol sulfate; (i)4-acetylcatechol sulfate (1)


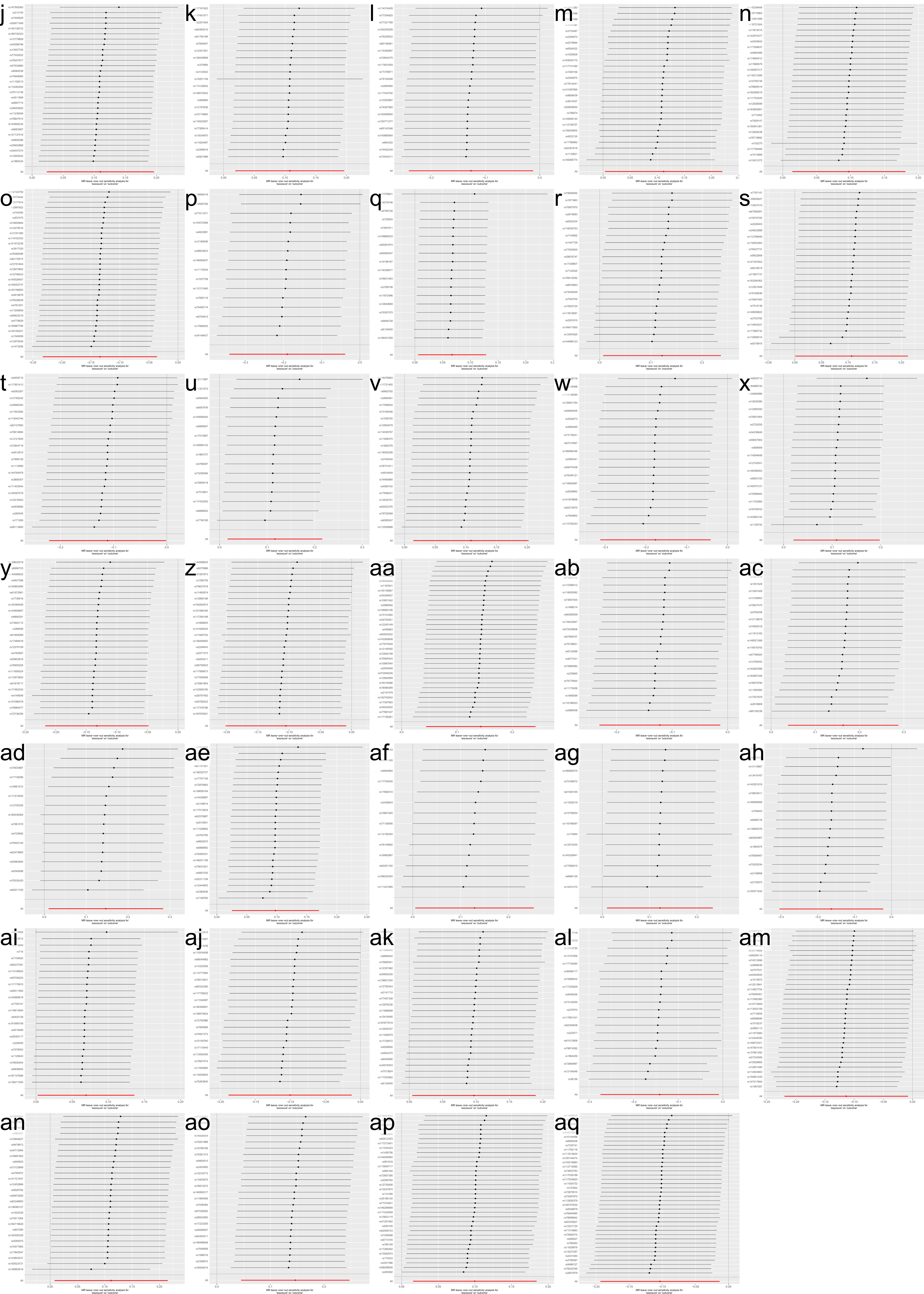


**Fig. S8: Leave-one-out plot for MR result of the serum metabolic and CP No2.**

(j)Taurochenodeoxycholic acid 3-sulfate; (k)N-acetyl-2-aminoadipate; (l)Eicosenedioate (C20:1-DC); (m)Glycerate; (n)Arachidonate (20:4n6); (o)Phenylpyruvate; (p)3-Hydroxybutyrate; (q)Malate; (r)Pentadecanoate (15:0); (s)X-11483; (t)X-12216; (u)X-12411; (v)X-12013; (w)X-13728; (x)X-14939; (y)X-17653; (z)X-21258; (a)X-21736; (b)X-22834; (c)X-25343; (d)X-25520; (e)N-acetylphenylalanine; (f)Adenosine 3',5'-cyclic monophosphate (cAMP) to taurocholate ratio; (g)Arachidonate (20:4n6) to pyruvate ratio; (h)Phosphate to fructose ratio; (i)Glutamine to asparagine ratio; (j)Spermidine to adenosine 5'-diphosphate (ADP) ratio; (k)Alpha-ketoglutarate to kynurenine ratio; (l)Ornithine to glutamate ratio; (m)Phosphate to tyrosine ratio; (n)Phenylpyruvate to 4-hydroxyphenylpyruvate ratio; (o)Tryptophan to pyruvate ratio; (p)Cholesterol to oleoyl-linoleoyl-glycerol (18:1 to 18:2) [2] ratio; (q)Glutamine to alanine ratio


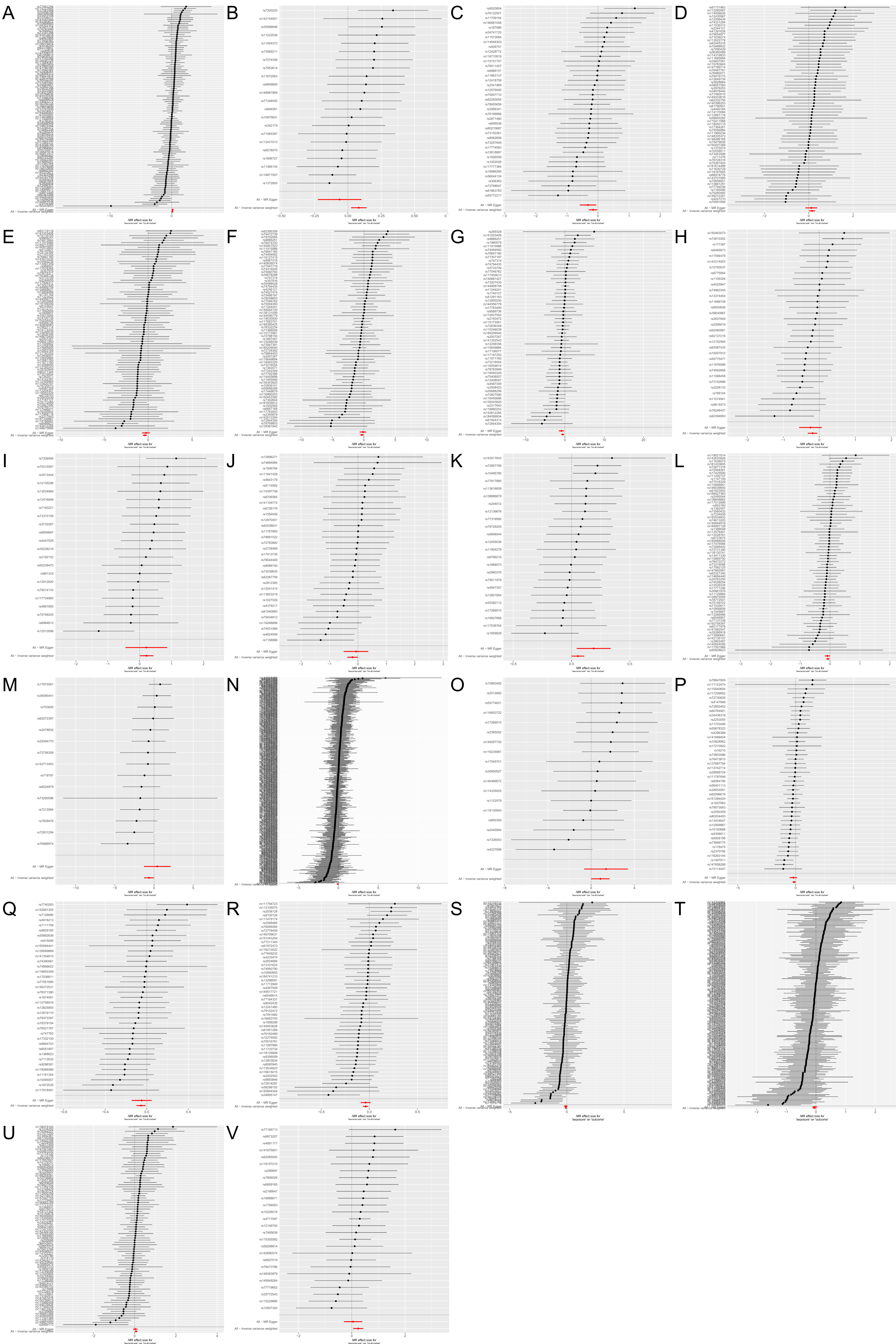


**Fig. S9: Forest for mr result of CSF metabolites and cp;**

(A)1,5-anhydroglucitol (1,5-ag); (B)Acetoacetate; (C)Alpha-ketoglutarate; (D)1-linoleoyl-gpc (18:2); (E)1-myristoyl-2-palmitoyl-gpc (14:0/16:0); (F)1-palmitoyl-2-oleoyl-gpc (16:0/18:1); (G)1-palmitoyl-2-palmitoleoyl-gpc (16:0/16:1); (H)2-hydroxyglutarate; (I)4-acetamidobutanoate; (J)Isoleucine; (K)Kynurenate; (L)Methionine sulfone; (M)O-sulfo-l-tyrosine; (N)Oxalate (ethanedioate); (O)Pseudouridine; (P)Pyridoxal; (Q)Spermidine; (R)X-12007; (S)Ascorbic acid 3-sulfate; (T)Butyrate (4:0); (U)X-23739; (V)X-24452;


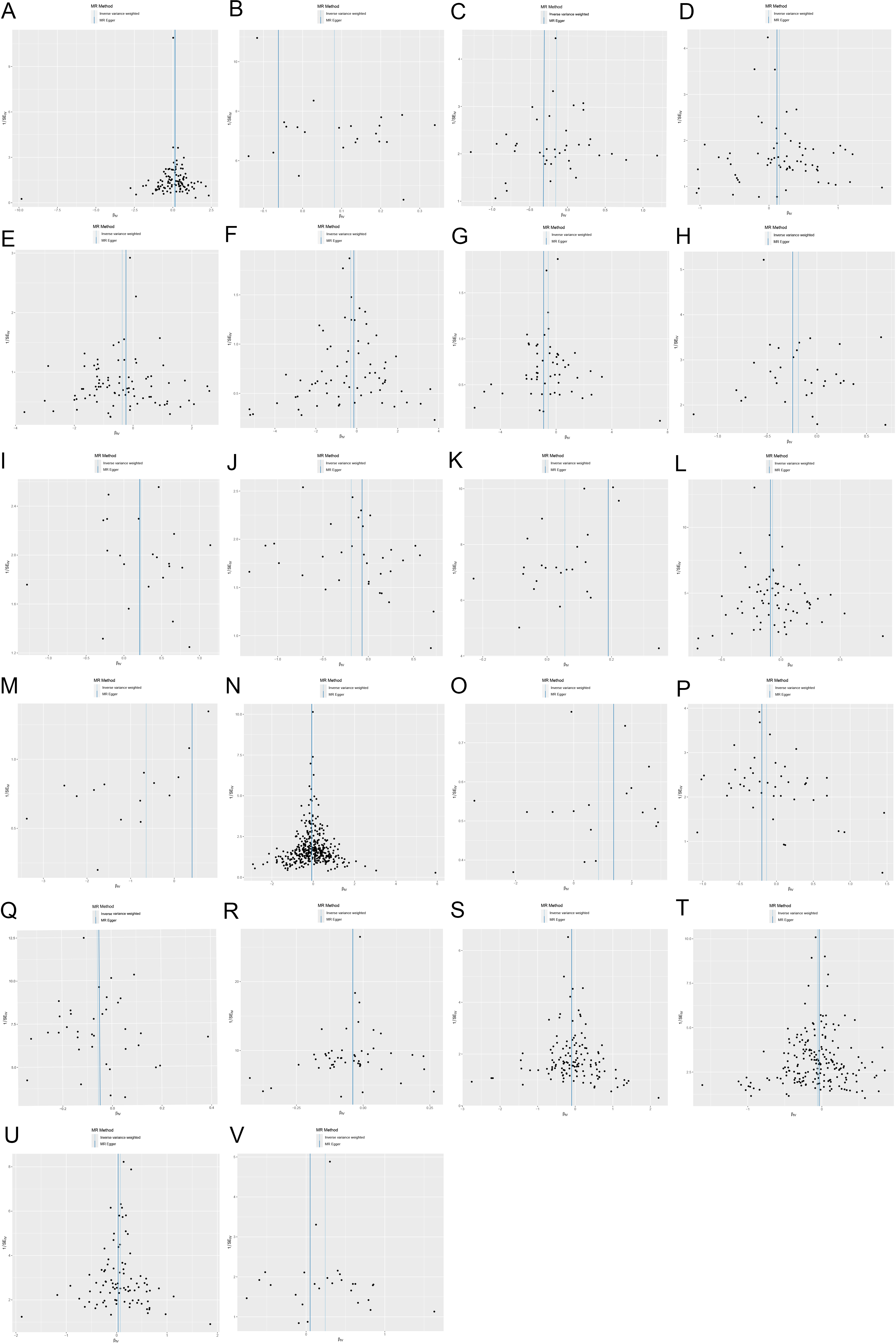


**Fig. S10: Funnel plot for MR result of CSF metabolites and CP;**

(A)1,5-anhydroglucitol (1,5-ag); (B)Acetoacetate; (C)Alpha-ketoglutarate; (D)1-linoleoyl-gpc (18:2); (E)1-myristoyl-2-palmitoyl-gpc (14:0/16:0); (F)1-palmitoyl-2-oleoyl-gpc (16:0/18:1); (G)1-palmitoyl-2-palmitoleoyl-gpc (16:0/16:1); (H)2-hydroxyglutarate; (I)4-acetamidobutanoate; (J)Isoleucine; (K)Kynurenate; (L)Methionine sulfone; (M)O-sulfo-l-tyrosine; (N)Oxalate (ethanedioate); (O)Pseudouridine; (P)Pyridoxal; (Q)Spermidine; (R)X-12007; (S)Ascorbic acid 3-sulfate; (T)Butyrate (4:0); (U)X-23739; (V)X-24452;


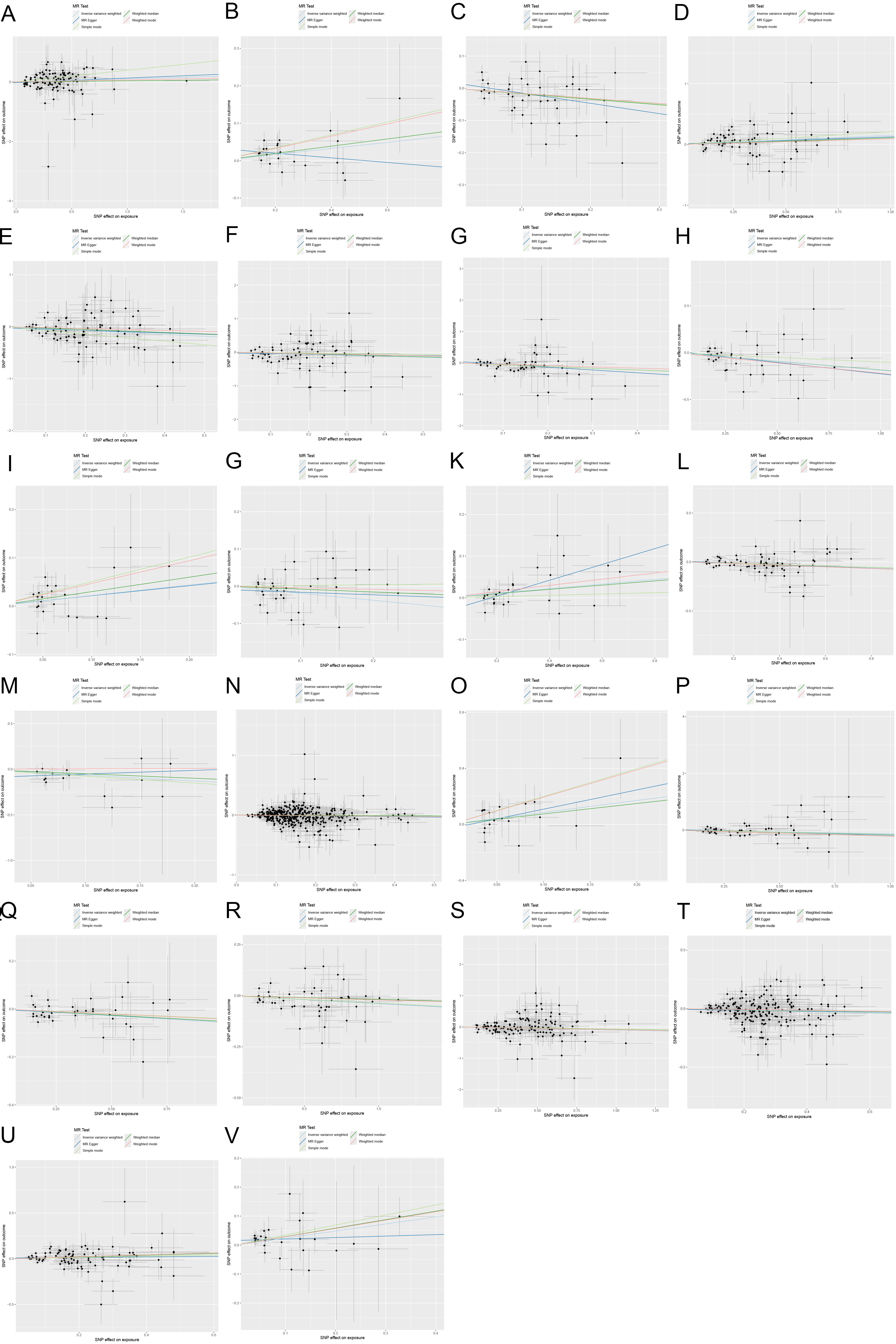


**Fig. S11: Scatter plot for MR result of the CSF metabolites and CP;**

(A)1,5-anhydroglucitol (1,5-ag); (B)Acetoacetate; (C)Alpha-ketoglutarate; (D)1-linoleoyl-gpc (18:2); (E)1-myristoyl-2-palmitoyl-gpc (14:0/16:0); (F)1-palmitoyl-2-oleoyl-gpc (16:0/18:1); (G)1-palmitoyl-2-palmitoleoyl-gpc (16:0/16:1); (H)2-hydroxyglutarate; (I)4-acetamidobutanoate; (J)Isoleucine; (K)Kynurenate; (L)Methionine sulfone; (M)O-sulfo-l-tyrosine; (N)Oxalate (ethanedioate); (O)Pseudouridine; (P)Pyridoxal; (Q)Spermidine; (R)X-12007; (S)Ascorbic acid 3-sulfate; (T)Butyrate (4:0); (U)X-23739; (V)X-24452;


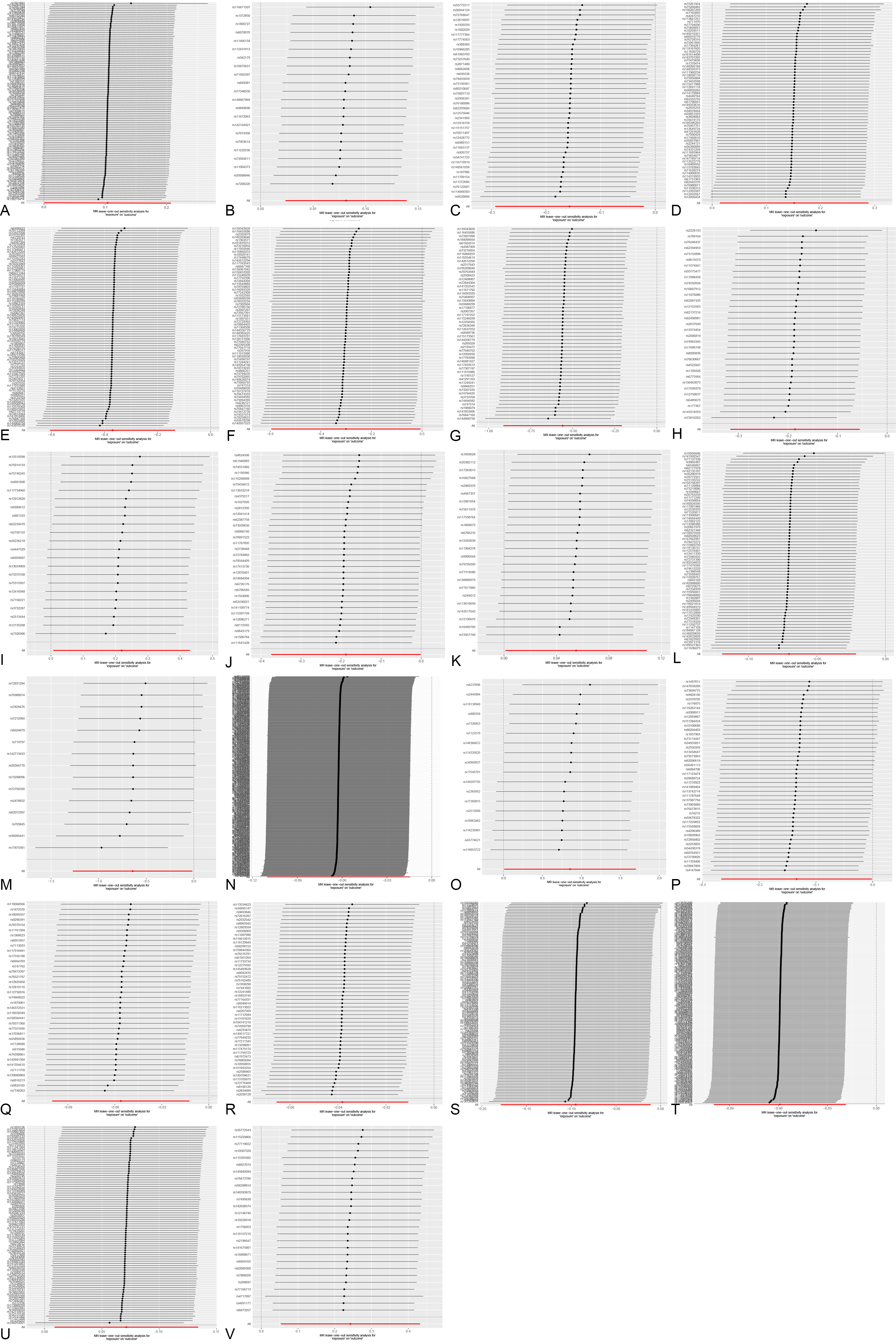


**Fig. S12: Leave one out for MR result of CSF metabolites and CP;**

(A)1,5-anhydroglucitol (1,5-ag); (B)Acetoacetate; (C)Alpha-ketoglutarate; (D)1-linoleoyl-gpc (18:2); (E)1-myristoyl-2-palmitoyl-gpc (14:0/16:0); (F)1-palmitoyl-2-oleoyl-gpc (16:0/18:1); (G)1-palmitoyl-2-palmitoleoyl-gpc (16:0/16:1); (H)2-hydroxyglutarate; (I)4-acetamidobutanoate; (J)Isoleucine; (K)Kynurenate; (L)Methionine sulfone; (M)O-sulfo-l-tyrosine; (N)Oxalate (ethanedioate); (O)Pseudouridine; (P)Pyridoxal; (Q)Spermidine; (R)X-12007; (S)Ascorbic acid 3-sulfate; (T)Butyrate (4:0); (U)X-23739; (V)X-24452;
